# Effectiveness of infection prevention and control interventions, excluding personal protective equipment, to prevent nosocomial transmission of SARS-CoV-2: a systematic review and call for action

**DOI:** 10.1016/j.infpip.2021.100192

**Published:** 2021-11-29

**Authors:** Yalda Jafari, Mo Yin, Cherry Lim, Diane Pople, Stephanie Evans, James Stimson, Thi Mui Pham, LSHTM CMMID COVID-19 working group, Jonathan M. Read, Julie V. Robotham, Ben S. Cooper, Gwenan M. Knight

**Affiliations:** a Centre for Mathematical Modelling of Infectious Diseases, IDE, EPH, London School of Hygiene & Tropical Medicine, London, United Kingdom; b Mahidol-Oxford Tropical Medicine Research Unit, Faculty of Tropical Medicine, Mahidol University, Bangkok, Thailand; c University Medicine Cluster, National University Hospital, Singapore; d Department of Medicine, National University of Singapore, Singapore; e Centre for Tropical Medicine, Nuffield Department of Medicine, University of Oxford, Oxford, UK; f Healthcare Associated Infections and Antimicrobial Resistance Division, National Infection Service, PHE, Colindale, London, UK; g Julius Center for Health Sciences and Primary Care, University Medical Center Utrecht, Utrecht University, Utrecht, the Netherlands; h Lancaster Medical School, Lancaster University, Lancaster, UK; i NIHR Health Protection Research Unit in Healthcare Associated Infections and Antimicrobial Resistance at University of Oxford in Partnership with Public Health England, Oxford, UK

## Abstract

Many infection prevention and control (IPC) interventions have been adopted by hospitals to limit nosocomial transmission of SARS-CoV-2. The aim of this systematic review is to identify evidence on the effectiveness of these interventions. We conducted a literature search of five databases (OVID MEDLINE, Embase, CENTRAL, COVID-19 Portfolio (pre-print), Web of Science). SWIFT ActiveScreener software was used to screen English titles and abstracts published between 1st January 2020 and 6th April 2021. Intervention studies, defined by Cochrane Effective Practice and Organisation of Care, that evaluated IPC interventions with an outcome of SARS-CoV-2 infection in either patients or healthcare workers were included. Personal protective equipment (PPE) was excluded as this intervention had been previously reviewed. Risks of bias were assessed using the Cochrane tool for randomised trials (RoB2) and non-randomized studies of interventions (ROBINS-I). From 23,156 screened articles, we identified seven articles that met the inclusion criteria, all of which evaluated interventions to prevent infections in healthcare workers and the majority of which were focused on effectiveness of prophylaxes. Due to heterogeneity in interventions, we did not conduct a meta-analysis. All agents used for prophylaxes have little to no evidence of effectiveness against SARS-CoV-2 infections. We did not find any studies evaluating the effectiveness of interventions including but not limited to screening, isolation and improved ventilation. There is limited evidence from interventional studies, excluding PPE, evaluating IPC measures for SARS-CoV-2. This review calls for urgent action to implement such studies to inform policies to protect our most vulnerable populations and healthcare workers.

## Introduction

Since the beginning of the COVID-19 pandemic, hospital-acquired SARS-CoV-2 infections have been reported across the world [[1], [2], [3], [4], [5]]. Healthcare workers are at higher risk of SARS-CoV-2 infection than the general population [6,7], in turn increasing the risk of transmission to their patients, co-workers and household members [6,8]. Hospitalised patients are often older and have more comorbidities than the general population [3] and hence are at a higher risk of becoming seriously ill [9].

SARS-CoV-2 infection is transmitted through close contact with an infected individual via droplet transmission, as well as via airborne and fomite transmission [10]. In healthcare settings, interventions such as mask wearing by patients and healthcare workers [[11], [12], [13]], screening of patients [[14], [15], [16]] and healthcare workers [[17], [18], [19]], and triaging of patients [20,21] have been implemented to reduce transmission of SARS-CoV-2. These interventions remain relevant despite emergence of new variants and uncertainties in effects of vaccines on transmissibility.

To determine the evidence for these interventions, we conducted a scoping review up to 28th January 2021 (Appendix A) for reviews of the effectiveness of IPC strategies to reduce SARS-CoV-2 transmission in hospital-based populations. We identified reviews of physical distancing [22], and masks [[22], [23], [24], [25], [26]] though none performed a meta-analysis due to a lack of intervention studies. Most notably, we identified a living rapid review by Chou *et al.* [23] reviewing the effectiveness of masks in health care and community settings in preventing transmission of respiratory viruses including SARS-CoV-2. Given the presence of such effort, we decided to exclude personal protective equipment (PPE) as part of our interventions under investigation. Instead, we focused on wider IPC measures, such as screening protocols, triaging, cohorting, ventilation, or physical barriers to transmission, for which we found no reviews.

Hence, the aim of this systematic review is to collate and assess evidence on the effectiveness of IPC interventions, excluding PPE, intended to reduce transmission of SARS-CoV-2 between patients, between patients and healthcare workers, and between healthcare workers within the hospital setting.

## Methods

We report the results of this systematic review following the guidelines of the Preferred Reporting Items for Systematic Reviews and Network Meta-Analyses (PRISMA) checklist [27].

### Protocol and registration

This systematic review is registered with the International Prospective Register of Systematic Reviews (PROSPERO) with the registration number CRD42021246617.

### Eligibility criteria

We included intervention studies as defined by the Cochrane Effective Practice and Organisation of Care (EPOC) group [28]. These studies include randomised trials, non-randomised trials, cluster randomised trials, repeated measures studies, interrupted time series studies, and controlled before-after studies. Observational studies were excluded.

We included any IPC strategy except personal protective equipment (PPE). PPE, as defined by the World Health Organisation in the context of COVID-19 includes medical masks, gloves, face shields, gowns, respirators (such as N95 or FFP2 standard or equivalent) and aprons [29]. Only studies that evaluated outcomes indicative of SARS-CoV-2 infections including clinical or laboratory confirmed diagnoses were included.

The population of interest included hospitalised patients and healthcare workers working in hospitals and thus were at risk of hospital-acquired SARS-CoV-2 infections. We included only English language studies.

We excluded studies that were published as letters, editorials, opinions, or brief communications.

### Information sources

Public Health England Knowledge and Learning Services conducted a search of Ovid Medline, Embase, CENTRAL, COVID-19 Portfolio (preprints), and Web of Science databases including studies between 1 January 2020 and 6 April 2021. The search strategy included terms to identify studies on SARS-CoV-2 infections, IPC, and the hospital setting. Each group of these terms was combined with an ‘AND’. A comprehensive search strategy is available in Appendix B.

### Study selection

We used the software SWIFT ActiveScreener [30] to screen titles and abstracts. Each record was reviewed by two reviewers split between four reviewers (CL, GK, MY, YJ). SWIFT ActiveScreener uses active learning to incorporate user feedback during the screening process and prioritize articles. A negative binomial model then identifies the number of relevant articles remaining [30]. Using this software, we reviewed a subset of articles until we reached a 95% estimated recall which is the probability of having included relevant articles. Conflicts were resolved by consensus between the two reviewers of each record.

Each included full text was reviewed by two reviewers split between four reviewers (CL, GK, MY, YJ) and conflicts were resolved by discussion.

### Data collection and data items

We collected information on study characteristics (publication status, study design), intervention characteristics, population characteristic (healthcare workers, patient, age, sex, co-morbidities), outcomes (clinical, laboratory based), and results (infections in study arms).

### Risk of bias in individual studies

Risk of bias in RCTs were assessed using the RoB2 tool [31]. The domains included risk of bias arising from the randomisation process, risk of bias due to deviations from the intended interventions, risk of bias due to missing outcome data, risk of bias in measurement of the outcome, risk of bias in selection of the reported result, followed by an overall risk of bias. A value judgement of low, high, or some risk of bias concern was assigned to each domain.

The risk of bias in non-RCTs were assessed using the ROBINS-I tool [32]. The ROBINS-I tool was used to assess bias due to confounding, bias in selection of participants into the study, bias in classifications of interventions, bias due to deviations from intended interventions, bias due to missing data, bias in measurements of outcomes, and bias in selection of the reported results, and an overall risk. A value judgement of low, moderate, serious, or critical risk of bias or no information was assigned to each domain.

Data from each article was extracted by one reviewer (YJ) and reviewed by a second reviewer (MY). Conflicts were resolved by discussion.

### Summary measures

We performed a descriptive analysis as the interventions were not comparable and were too few to conduct a meta-analysis.

## Results

**Figure 1:**
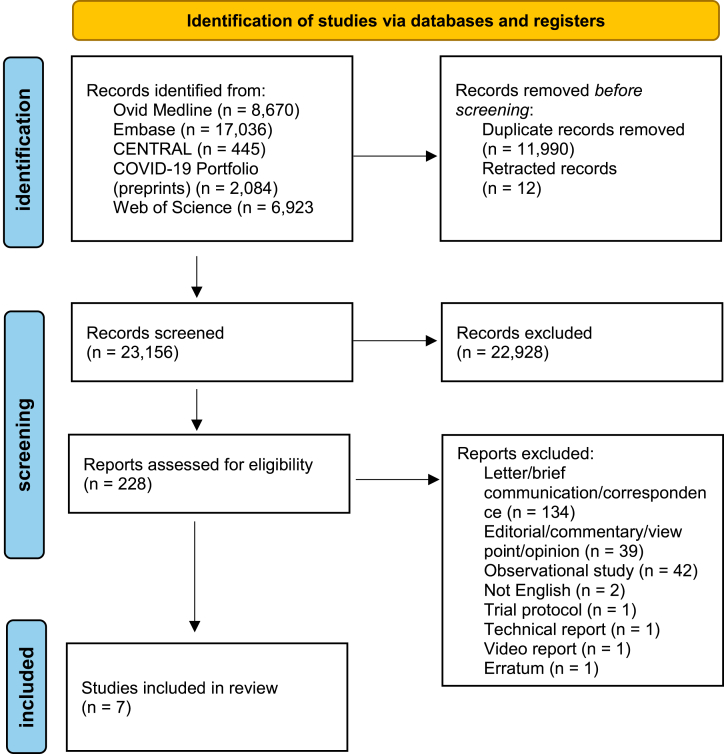
Study selection.

Our initial search identified 35,158 records (Figure 1). After de-duplication, 23,711 records were identified and uploaded to SWIFTActive Screener. We (CL, MY, GK, YJ) double screened 8,807 (37%) records, and reached 97% recall. Through this process, 238 records which matched our search criteria were identified, as well as 531 more duplicates identified during the review process resulting in a final of 228 full records to be reviewed. Of these, seven records met the inclusion criteria (Figure 1).

Six of these studies were randomised control trials while one was a non-randomised trial. Studies were conducted in Argentina [33], Canada [34], India [35], Pakistan [36], Spain [37], and the USA [34,38]. All studies evaluated efficacy of interventions in healthcare workers only, with no studies conducted in patient populations.

Six studies evaluated the use of pre-exposure prophylaxes (hydroxychloroquine [34,37,38], bromhexine hydrochloride [39], ivermectin and iota-carrageenan [33], chyawanprash (a herbal supplement) [35]), while one study [36] evaluated the effectiveness of an audio-visual triage system.

We found no studies which evaluated the effectiveness of other widely used strategies including isolation, cohorting of patients and staff, improved ventilation strategies, use of air filters, or enhanced environmental cleaning.

### Summary of studies

Studies did not have comparable interventions, and hence a meta-analysis was not conducted. Summary details of studies are presented in Table I, with a more detailed extraction in Appendix C.

**Table I:** Characteristics of included studies

Study	Type of study	No. of participants	Age	% female	Co-morbidities	Hospital setting	Baseline IPC measures	Type of intervention	Intervention description	Duration of follow up	Control	Primary outcome	Secondary outcome
Abella 2020	RCT	132	Median: 33 years (range, 20–66)	69%	Asthma (17%), Diabetes (3%), Hypertension (21%)	Emergency department, dedicated COVID-19 units	Use of PPE (including masks, eyewear, and gowns) as well as patient screening for COVID-19 symptoms	Pre-exposure prophylaxis	Hydroxychloroquine 200-mg tablets, 3 tablets once a day	8 wks	Placebo	Rate of conversion to SARS-CoV-2 positive status via NP RT-PCR after 8 weeks of treatment	Adverse event rate; rate of serologic antibody positivity for either nucleocapsid or spike protein antigens; ECG changes after 4 weeks of treatment; clinical outcomes for any partici- pants who became SARS-CoV-2 positive and/or developed COVID-19 symptoms within study period.
Chahla, 2021	RCT	234	Median: 38 years (min: 22; max: 69)	57.30%	Hypertension (9%), Diabetes (7%), Obesity (12%), >60 years (4%), Renal (2%)	Healthcare centres	standard biosecurity care and personal protective equipment (PPE).	Pre-exposure prophylaxis	Ivermectin (2 tablets of 6 mg weekly) and Iota-Carrageenan (6 sprays per day)	4 wks	Standard biosecurity care and personal protective equipment (PPE).	Reduction in COVID-19 disease rate, measured by RT-PCR	Reduction in presence of COVID-19 symptoms; protection against the appearance of severe stages for COVID-19 disease
Grau-Pujol, 2021	RCT	269	Median: 39 years (IQR: 30–50 years)	73%	Diabetes (0.4%), Hypertension (1.9%), Chronic respiratory condition (2.6%), Other(27.9%)	Hospital, specific unit unclear.	83% always used COVID-19 recommended PPE at work during the last 20 days	Pre-exposure prophylaxis	Hydroxychloroquine (2 tablets of 200 mg daily for first 4 days, then 400mg weekly)	6 mos	Placebo	Incidence of compatible symptoms with COVID-19 with seroconversion or a positive RT-PCR between study arms	the SARS-CoV-2 seroconversion in study arms in both asymptomatic and symptomatic participants; adverse events (AE) related to hydroxychloroquine treatment; incidence of SARS-CoV-2 infection in placebo group; risk ratio for the different clinical, analytical and microbiological conditions to develop COVID-19.
Gupta, 2021	RCT	199	Intervention: mean: 32.1 (SD:7.4); Control: Mean: 33.6 (SD: 8.6)	Intervention: 40.8% out of 98, Control: 50.5% out of 95	Malnourished (3.1%)	COVID-19 isolation ward	Standard Preventive Regimen as per institutional guidelines and based on roles	Pre-exposure prophylaxis	Chyawanprash (12 g twice daily)	30 days	Standard preventive regimen	Incidence of COVID-19 cases in both groups confirmed by RT-PCR	Comparing the biochemical and hematological parameters before and after the study and through occurrence of any adverse drug reactions; assessment of efficacy of Chyawanprash in preventing other infective diseases through incidence of symptoms; evaluation of effect of Chyawanprash on immunoglobulins and inflammatory markers through comparing the levels of IgG, IgM, IgE, high sensitivity C-Reactive Protein (hsCRP), Tumor Necrosing Factor alpha (TNF alpha) and Interleukins viz., IL-6 and IL-10.
Mikhaylov, 2021	RCT	50	Mean: 40.6 years (SD: 7.6)	58%	Hypertensive (6%); Hypercholesterolemia (4%)	Emergency departments where patients with confirmed/suspected COVID-19 were admitted, intensive care units, and clinical departments	PPE as prescribed by WHO recommendations and local instructions. PPE included respirators class FFP2 or FFP3, full skin covering, and protective eyeglasses.	Pre-exposure prophylaxis	Bromhexine hydrochloride treatment (8 mg 3 times per day)	8 wks	Standard care	positive nasopharyngeal swab SARS-CoV-2 PCR test or the presence of clinical symptoms of infection within 28 days and during the weeks 5–8 after the last contact to subjects with COVID-19	Time from the first contact with a person with suspected/confirmed COVID-19 to the appearance of respiratory infection symptoms; number of days before first positive SARS-CoV-2 test; number of asymptomatic participants with a positive nasopharyngeal swab test; the number of mild, moderate and severe COVID-19 cases;
Rajasingham, 2020	RCT	1483	Median: 41 years (interquar- tile range [IQR], 34 to 49)	51%	Hypertension (14%), Asthma (10%),	Emergency department or intensive care unit, on a dedicated COVID-19 hospital ward	Mask/faceshield use reported over 80% in all groups	Pre-exposure prophylaxis	Hydroxychlroquine (400 mg (2 200-mg tablets) twice separated by 6–8 hours followed by [1] 400 mg (2 200-mg tablets) once weekly or [2] 400 mg (2 200-mg tablets) twice weekly	12 wks	Placebo	COVID-19–free survival time by PCR confirmed or probable compatible illness.	incidence of confirmed SARS-CoV-2 detection; incidence of possible COVID-19; incidence of hospitalization, death, or other adverse events.
Hafeez, 2020	non-RT	60	Intervention: Min-max (20–35); Control: Min-max (20–38)	28.30%	Anxiety (93.3%)	Entrance of the hospital	Standard PPE (did not specify what types)	Audio-visual triage	Glass barrier sheet at triage desk at a distance of more than 6 feet from patient desk, both desks connected with non-touchable mic system for communication	1 wk	Visual triage (outside at entry door)	Anxiety levels	COVID-19 PCR results

Number of participants in RCTs ranged from 50 to 1483. Abella *et al.* [38], Chahla *et al.* [33], Grau-pujol *et al.* [37], and Gupta *et al.* [35] used polymerase chain reaction (PCR) tests to identify SARS-CoV-2 infections while Mikhaylov *et al.* [39] and Rajasingham *et al.* [34] used a combination of laboratory tests and clinical symptoms.

All three RCTs evaluating hydroxychloroquine [34,37,38] were stopped early as they were underpowered to detect any clinical significance. The trial evaluating chyawanprash [35] was also underpowered. The RCT evaluating bromhexine hydrochloride [39] did not find statistically significant results between the two arms (intervention: 2/25 (8%), control group: 7/25 (28%), *P* = 0.07) while the RCT evaluating ivermectin and iota-carrageenan [33] found a protective effect of the intervention (intervention: 4/117 (3.4%), control: 25/117 (21.4%), p=1x10^−5^).

A non-randomised trial [36] evaluated the effectiveness of an audio-visual triage system separating the patient and staff by a glass barrier, at a distance of six feet, and connected by an audio system. Sixty staff were enrolled, with a PCR test used to identify SARS-CoV-2 infections. The authors found a statistically significant lower rate of infection in the intervention group (intervention: 3/30 (10%), control: 9/30 (30%), *P*=0.001).

Two studies [34,38] noted that authors received income from pharmaceutical companies, not funding the work related to the studies. In one study [37], a pharmaceutical company partly funded the study while in another study [35] it was unclear whether a drug under investigation was donated. One study [36] did not include a statement on conflict of interest while another [33] did not identify any conflicts of interest.

### Risk of bias

**Figure 2:**
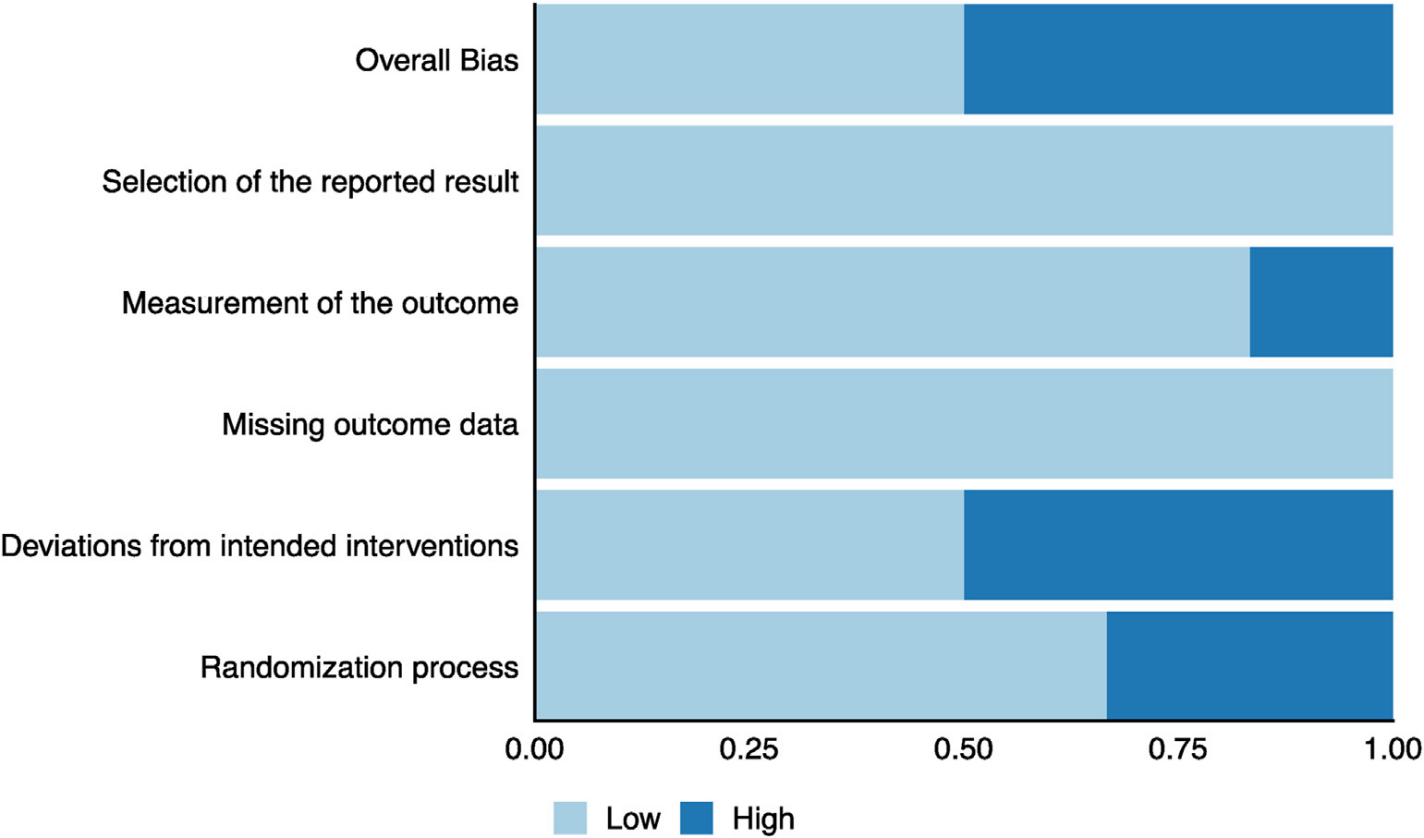
Risk of bias of included RCTs. Horizontal axis shows proportion of studies with high risk of bias and vertical axis indicates each domain of bias assessed.

Using RoB2 for the RCTs, two studies [33,39] had a high risk of bias due to randomisation process, while half had a high risk of bias due to deviation from intended interventions [28,32,33] (Figure 2). All studies had a low risk of bias due to missing outcome data. One study [39] had a high risk of bias in the measurement of the outcome, while all studies had a low risk of bias of the selection of the reported results. Three studies [33,35,39] had a high overall risk of bias. Results of individual studies assessed using the RoB2 are presented in Table II.

**Table II:** ROB2 results of individual studies

Study	Intervention	Control	Outcome	Randomisation process	Deviations from the intended interventions	Missing outcome data	Measurement of the outcome	Selection of the reported result	Overall risk of bias all
Abella *et al.*	Hydroxychloroquine	Placebo	Positive PCR	Low	Low	Low	Low	Low	Low
Chahla *et al.*	Ivermectin/Iota-Carrageenan (IVER/IOTACRC)	None	Positive PCR	High	High	Low	Low	Low	High
Grau-Pujol *et al.*	Hydroxychloroquine	Placebo	Symptoms with seroconversion or a positive PCR	Low	Low	Low	Low	Low	Low
Gupta *et al.*	Chyawanprash	None	Positive PCR	Low	High	Low	Low	Low	High
Mikhaylov *et al.*	Bromhexine hydrochloride	None	Symptoms or positive PCR	High	High	Low	High	Low	High
Rajasingham *et al.*	Hydroxychloroquine	Placebo	Positive PCR	Low	Low	Low	Low	Low	Low

Using the ROBINS-I tool to assess the risk of bias in one included non-RCT [36], the risk of bias was considered moderate for confounding and selection bias, and low risk for classification of interventions, reporting bias, deviations from interventions, missing data, and measuring outcomes. Overall risk of bias was moderate. More detail can be found in Table III.

**Table III:** ROBINS results of individual studies

Study	Intervention	Control	Outcome	Confounding	Selection Bias	Classification of interventions	Reporting Bias	Deviations from interventions	Missing data	Measuring outcomes	Overall
Hafeez *et al.*	Audio-visual system	Visual system	Positive PCR	Moderate	Moderate	Low	Low	Low	Low	Low	Moderate

## Discussion

We found seven studies matching our inclusion criteria, of which six focused on evaluating prophylactics and one study evaluated an audio-visual system during triage. None of the six proposed agents have good evidence for efficacy in preventing infection with SARS-CoV-2 [40]. Notably, we did not find any studies with appropriate study design for assessing interventions proposed by international [10,41] and national [[42], [43], [44], [45]] guidelines and adopted by some hospitals, including cohorting, screening, isolating, use of single rooms, and use of environmental and engineering strategies such as improved ventilation and use of filters. Similarly to a review on the effectiveness of ultraviolet-C (UV–C) in hospitals [39], we did not find any studies using UV-C or any other sterilisation strategies to prevent transmission of SARS-CoV-2. Consequently, generating high quality evidence for IPC against nosocomial transmission of SARS-CoV-2 continues to be a priority, particularly for costly interventions or interventions that have the potential to cause harm such as mass administration of prophylaxis to healthcare workers [46].

All identified studies only included healthcare workers, and the majority evaluated pharmaceutical interventions. Three studies evaluating hydroxychloroquine did not find a prophylactic effect and were stopped early. This was similar to the findings of Lewis *et al.* [18,19] and Bartoszko *et al.* [22] which reviewed effectiveness of hydroxychloroquine as prophylaxis though not specifically in a hospital setting. Bartoszko *et al.* [18], through their systematic review of effectiveness of prophylaxes for prevention of SARS-CoV-2 infections in all settings, included the same trial on the evaluation of ivermectin and iota-carrageenan as found here. Similar to their findings, we found that despite the demonstrated impact of ivermectin and iota-carrageenan as a prophylaxis to reduce SARS-CoV-2 transmission, the quality of evidence was low due to high risk of bias, mainly due to the lack of blinding. Studies evaluating bromhexine hydrochloride [39] and chyawanprash [35] also suffered from high bias due to lack of blinding of interventions.

In the non-RCT, non-pharmaceutical intervention trial that evaluated an audio-visual system [36], the authors found a significant decrease in infections in the intervention arm. There was, however, a moderate risk of confounding with analysis only including a small number of covariates and selection bias with a poor description of recruitment strategy. Hence, this evidence does not support implementation of the evaluated system.

Chou *et al.* [23] last updated their live systematic review on effectiveness of respirators, face masks, and cloth masks to prevent SARS-CoV-2 infections in healthcare settings in July 2021 [47]. Their inclusion criteria included RCT and observational studies. They did not identify any RCTs through their search but identified ten observational studies. However, due to methodological limitations of the studies and heterogeneity of comparisons and results, meta-analysis was not conducted. Therefore, evidence on effectiveness of masks to prevent transmission of SARS-CoV-2 in healthcare settings remains insufficient.

Since there is limited evidence to guide hospital practice for the prevention of nosocomial transmission of SARS-CoV-2, learnings can be taken from studies collating evidence on the effectiveness of interventions in preventing hospital-acquired infections of other respiratory viruses. A review on infection and prevention strategies to prevent seasonal influenza [42] found limited evidence to support screening HCWs, patients, and visitors and isolation of infectious individuals. Evidence of intervention strategies for other types of hospital-acquired infections can also be informative. A review [48] of the effectiveness of cohorting in reducing transmission of *C. difficile* and multi-drug organisms found limited evidence in its effectiveness despite its widespread use.

This review highlights the lack of available evidence on the effectiveness of IPC strategies for SARS-CoV-2 infection in hospitals indicating further research is needed. This could be a result of increased workload of hospital and public health staff, inadequate understanding of transmission dynamics of the virus in the hospital setting, and challenges in implementing IPC interventional studies (e.g., many interventions involve behaviour change which is difficult to attain and assess, institutional commitment for systemic change, interventions are usually executed in bundles which are resource-intensive). These may have prevented timely and appropriate implementation of important clinical research to assess interventions to inform practice.

With an increased understanding of SARS-CoV-2 transmission and disease and more managed workload in many settings, there should be a renewed focus on evaluating IPC practices. For example, interventions to be investigated include type and frequency of patient and HCW screening, isolation practices, cohorting strategies of both patients and HCWs, improved ventilation and methods for air circulation, and environmental cleaning. Many of these are already suggested as part of the infection prevention and control recommendations by Public Health England [49]. The importance of SARS-CoV-2 vaccination status for both patients and HCWs could also be investigated and would be an important confounder in any future intervention study.

Furthermore, enhancing national and local guidelines on implementing interventional studies to evaluate IPC practices would greatly improve the feasibility, ease, and timeliness of their implementations and ensure appropriateness of studies particularly when such research may not be deemed a priority. Researchers should follow the Standards for Reporting Implementation Studies (StaRI) Statement and Checklist [50], which applies to a range of study designs, to help clarify planning and implementation and to ensure accurate and transparent reporting of studies. The StaRI statement consists of a checklist of 27 items, describing both the implementation strategy and the implemented intervention.

There were some limitations with our review. Firstly, we only included English language studies. The COVID-19 pandemic has occurred in every country in the world, each with their unique healthcare delivery system, practices, and challenges. By excluding non-English studies, we may have missed studies with transferrable evidence between settings. Secondly, we did not include studies evaluating the effectiveness of PPE interventions. Some of these studies, however, may have evaluated other interventions along with PPE but may not have explicitly identified them in keywords or abstract. Thirdly, due to the large number of identified records, we used a software, SWIFT-Active Screener, to screen titles and abstracts until we reached a recall probability of 97%. We may have missed some studies by not reaching 100% recall or reviewing all references. However, using this software greatly reduced the effort required to conduct this systematic review and we are confident that we would not have found any additional relevant articles [30].

## Conclusions

There is currently very little evidence available on the effectiveness of interventions, excluding PPE, to prevent the spread of SARS-CoV-2 in hospital settings. Our systematic review revealed no appropriate studies specifically targeting prevention of transmission between hospitalised patients. While this likely reflects the pressures health systems have been, and are, under, and that many logical, likely effective IPC measures are in place, without unbiased, intervention studies we cannot say which are optimal and cannot maximise the protection given to our most vulnerable populations and those that care for them. This review underscores the need to generate evidence for IPC interventions in hospitals to prevent transmission of SARS-CoV-2, which could also be applicable to other pathogens and calls for high quality intervention studies to systematically determine which IPC measures to implement in healthcare settings.
