# Supplementary material for: Effectiveness of infection prevention and control interventions, excluding personal protective equipment, to prevent nosocomial transmission of SARS-CoV-2: a systematic review and call for action
Source: Infect Prev Pract. 2021 Nov 29;4(1):100192. doi: 10.1016/j.infpip.2021.100192 (PMC8628369; doi:10.1016/j.infpip.2021.100192)
Supplement: Multimedia component 1 [file mmc1.docx]

**Appendix A: Sources used for rapid review conducted on 28^th^ January 2021**

| UNCOVER (Usher Network for Covid-19 Evidence Reviews) | [https://www.ed.ac.uk/usher/uncover/register-of-reviews](https://eur01.safelinks.protection.outlook.com/?url=https%3A%2F%2Fwww.ed.ac.uk%2Fusher%2Funcover%2Fregister-of-reviews&data=04%7C01%7CRachel.Gledhill%40phe.gov.uk%7C858df0dbbf8b42896a8608d941412c7f%7Cee4e14994a354b2ead475f3cf9de8666%7C0%7C0%7C637612570432097161%7CUnknown%7CTWFpbGZsb3d8eyJWIjoiMC4wLjAwMDAiLCJQIjoiV2luMzIiLCJBTiI6Ik1haWwiLCJXVCI6Mn0%3D%7C1000&sdata=DBfDXi6FIlEt8g%2FB6cv3I67EaBIe1BAgaYxv%2Bz1YQEc%3D&reserved=0) |
| --- | --- |
| Epistemonikos, Covid-19 L.ove | [https://app.iloveevidence.com/loves/5e6fdb9669c00e4ac072701d](https://eur01.safelinks.protection.outlook.com/?url=https%3A%2F%2Fapp.iloveevidence.com%2Floves%2F5e6fdb9669c00e4ac072701d&data=04%7C01%7CRachel.Gledhill%40phe.gov.uk%7C858df0dbbf8b42896a8608d941412c7f%7Cee4e14994a354b2ead475f3cf9de8666%7C0%7C0%7C637612570432097161%7CUnknown%7CTWFpbGZsb3d8eyJWIjoiMC4wLjAwMDAiLCJQIjoiV2luMzIiLCJBTiI6Ik1haWwiLCJXVCI6Mn0%3D%7C1000&sdata=Vek5%2Bs7kNJpeHpvDGlnS55%2B3CTEkVETwq%2B25LSYQPuY%3D&reserved=0) |
| VA Evidence Synthesis Program | [http://covid19reviews.org/](https://eur01.safelinks.protection.outlook.com/?url=http%3A%2F%2Fcovid19reviews.org%2F&data=04%7C01%7CRachel.Gledhill%40phe.gov.uk%7C858df0dbbf8b42896a8608d941412c7f%7Cee4e14994a354b2ead475f3cf9de8666%7C0%7C0%7C637612570432107123%7CUnknown%7CTWFpbGZsb3d8eyJWIjoiMC4wLjAwMDAiLCJQIjoiV2luMzIiLCJBTiI6Ik1haWwiLCJXVCI6Mn0%3D%7C1000&sdata=y992DvGLQMFC3uiqeRF7Uq7bMYHLH5Q7%2BpqyVxQYd%2Fw%3D&reserved=0) |
| COVID-19 Best Evidence Front Door, University of Michigan | [https://frontdoor.knack.com/covidbestevidence/](https://eur01.safelinks.protection.outlook.com/?url=https%3A%2F%2Ffrontdoor.knack.com%2Fcovidbestevidence%2F&data=04%7C01%7CRachel.Gledhill%40phe.gov.uk%7C858df0dbbf8b42896a8608d941412c7f%7Cee4e14994a354b2ead475f3cf9de8666%7C0%7C0%7C637612570432107123%7CUnknown%7CTWFpbGZsb3d8eyJWIjoiMC4wLjAwMDAiLCJQIjoiV2luMzIiLCJBTiI6Ik1haWwiLCJXVCI6Mn0%3D%7C1000&sdata=02n3zHq0ZKP1EeF7y0sEi4XDsJSDrjw%2FLwHM9JvQF2k%3D&reserved=0) |
| COVID-END Evidence about public-health measures | [https://www.mcmasterforum.org/networks/covid-end/resources-to-support-decision-makers/Inventory-of-best-evidence-syntheses/public-health-measures](https://eur01.safelinks.protection.outlook.com/?url=https%3A%2F%2Fwww.mcmasterforum.org%2Fnetworks%2Fcovid-end%2Fresources-to-support-decision-makers%2FInventory-of-best-evidence-syntheses%2Fpublic-health-measures&data=04%7C01%7CRachel.Gledhill%40phe.gov.uk%7C858df0dbbf8b42896a8608d941412c7f%7Cee4e14994a354b2ead475f3cf9de8666%7C0%7C0%7C637612570432117075%7CUnknown%7CTWFpbGZsb3d8eyJWIjoiMC4wLjAwMDAiLCJQIjoiV2luMzIiLCJBTiI6Ik1haWwiLCJXVCI6Mn0%3D%7C1000&sdata=981D0jkqYOHt78xLPFkHS%2FUoTmhWVeOU%2B78FT5qj4iE%3D&reserved=0) |
| Health Information and Quality Authority, Ireland | [https://www.hiqa.ie/areas-we-work/health-technology-assessment/covid-19-publications](https://eur01.safelinks.protection.outlook.com/?url=https%3A%2F%2Fwww.hiqa.ie%2Fareas-we-work%2Fhealth-technology-assessment%2Fcovid-19-publications&data=04%7C01%7CRachel.Gledhill%40phe.gov.uk%7C858df0dbbf8b42896a8608d941412c7f%7Cee4e14994a354b2ead475f3cf9de8666%7C0%7C0%7C637612570432117075%7CUnknown%7CTWFpbGZsb3d8eyJWIjoiMC4wLjAwMDAiLCJQIjoiV2luMzIiLCJBTiI6Ik1haWwiLCJXVCI6Mn0%3D%7C1000&sdata=q%2FWfkjTbzP9HumQJ%2FbF%2F8tYuqP%2B9fE3JuZvgZLVdQOs%3D&reserved=0) |
| McMaster Uni, National Collaborating Centre for Methods and Tools (NCCMT) | [https://www.nccmt.ca/knowledge-repositories/covid-19-evidence-reviews](https://eur01.safelinks.protection.outlook.com/?url=https%3A%2F%2Fwww.nccmt.ca%2Fknowledge-repositories%2Fcovid-19-evidence-reviews&data=04%7C01%7CRachel.Gledhill%40phe.gov.uk%7C858df0dbbf8b42896a8608d941412c7f%7Cee4e14994a354b2ead475f3cf9de8666%7C0%7C0%7C637612570432127034%7CUnknown%7CTWFpbGZsb3d8eyJWIjoiMC4wLjAwMDAiLCJQIjoiV2luMzIiLCJBTiI6Ik1haWwiLCJXVCI6Mn0%3D%7C1000&sdata=bzsi0OA%2F0FdVJWGyt%2BiEwIp8BIJ9GpROIQ%2BjQmUi%2B1E%3D&reserved=0) |
| SPOR | [https://sporevidencealliance.ca/test-2/covid-19-evidence-synthesis/](https://eur01.safelinks.protection.outlook.com/?url=https%3A%2F%2Fsporevidencealliance.ca%2Ftest-2%2Fcovid-19-evidence-synthesis%2F&data=04%7C01%7CRachel.Gledhill%40phe.gov.uk%7C858df0dbbf8b42896a8608d941412c7f%7Cee4e14994a354b2ead475f3cf9de8666%7C0%7C0%7C637612570432127034%7CUnknown%7CTWFpbGZsb3d8eyJWIjoiMC4wLjAwMDAiLCJQIjoiV2luMzIiLCJBTiI6Ik1haWwiLCJXVCI6Mn0%3D%7C1000&sdata=Xey%2B418DIRsZP4l5L9pdvRIKXbKC%2F2zHZimy9p840Qw%3D&reserved=0) |
| LitCOVID | [https://www.ncbi.nlm.nih.gov/research/coronavirus/](https://eur01.safelinks.protection.outlook.com/?url=https%3A%2F%2Fwww.ncbi.nlm.nih.gov%2Fresearch%2Fcoronavirus%2F&data=04%7C01%7CRachel.Gledhill%40phe.gov.uk%7C858df0dbbf8b42896a8608d941412c7f%7Cee4e14994a354b2ead475f3cf9de8666%7C0%7C0%7C637612570432136992%7CUnknown%7CTWFpbGZsb3d8eyJWIjoiMC4wLjAwMDAiLCJQIjoiV2luMzIiLCJBTiI6Ik1haWwiLCJXVCI6Mn0%3D%7C1000&sdata=EjTTPSqtJiPFk3X8tqBB6aohafaQzLv0b9H3JLH0T4Y%3D&reserved=0) |
| NLM Covid portfolio | [https://icite.od.nih.gov/covid19/search/](https://eur01.safelinks.protection.outlook.com/?url=https%3A%2F%2Ficite.od.nih.gov%2Fcovid19%2Fsearch%2F&data=04%7C01%7CRachel.Gledhill%40phe.gov.uk%7C858df0dbbf8b42896a8608d941412c7f%7Cee4e14994a354b2ead475f3cf9de8666%7C0%7C0%7C637612570432136992%7CUnknown%7CTWFpbGZsb3d8eyJWIjoiMC4wLjAwMDAiLCJQIjoiV2luMzIiLCJBTiI6Ik1haWwiLCJXVCI6Mn0%3D%7C1000&sdata=TgLB6pHDyL46UGwLrQd6d%2B55E%2BlPBvDuDPkFNImq44g%3D&reserved=0) |
| Cochrane | [https://covidrapidreviews.cochrane.org/](https://eur01.safelinks.protection.outlook.com/?url=https%3A%2F%2Fcovidrapidreviews.cochrane.org%2F&data=04%7C01%7CRachel.Gledhill%40phe.gov.uk%7C858df0dbbf8b42896a8608d941412c7f%7Cee4e14994a354b2ead475f3cf9de8666%7C0%7C0%7C637612570432136992%7CUnknown%7CTWFpbGZsb3d8eyJWIjoiMC4wLjAwMDAiLCJQIjoiV2luMzIiLCJBTiI6Ik1haWwiLCJXVCI6Mn0%3D%7C1000&sdata=mGiljWxHZrWx%2BaC3tSxCzivuapHw3pldR1Ckf0L81Hw%3D&reserved=0) |
| Emergency Care Research Insititute (ECRI) | [https://www.ecri.org/covid-19-clinical-evidence-assessments](https://eur01.safelinks.protection.outlook.com/?url=https%3A%2F%2Fwww.ecri.org%2Fcovid-19-clinical-evidence-assessments&data=04%7C01%7CRachel.Gledhill%40phe.gov.uk%7C858df0dbbf8b42896a8608d941412c7f%7Cee4e14994a354b2ead475f3cf9de8666%7C0%7C0%7C637612570432146947%7CUnknown%7CTWFpbGZsb3d8eyJWIjoiMC4wLjAwMDAiLCJQIjoiV2luMzIiLCJBTiI6Ik1haWwiLCJXVCI6Mn0%3D%7C1000&sdata=sDK891%2Bqgi7E%2FRec%2Bd3UO%2F5Q5JAuP2BI28%2F2ofLRc%2FE%3D&reserved=0) |
| Lenus, The Irish Health Repository, Covid-19 Rapid Reviews & Evidence Summaries | [https://www.lenus.ie/handle/10147/627286](https://eur01.safelinks.protection.outlook.com/?url=https%3A%2F%2Fwww.lenus.ie%2Fhandle%2F10147%2F627286&data=04%7C01%7CRachel.Gledhill%40phe.gov.uk%7C858df0dbbf8b42896a8608d941412c7f%7Cee4e14994a354b2ead475f3cf9de8666%7C0%7C0%7C637612570432146947%7CUnknown%7CTWFpbGZsb3d8eyJWIjoiMC4wLjAwMDAiLCJQIjoiV2luMzIiLCJBTiI6Ik1haWwiLCJXVCI6Mn0%3D%7C1000&sdata=krThu1rybwI070A7t%2FA6htvuHpuuAfPDy3Isn1a5CMM%3D&reserved=0) |
| National Institute for Health and Care Excellence (NICE) | [https://www.nice.org.uk/covid-19](https://eur01.safelinks.protection.outlook.com/?url=https%3A%2F%2Fwww.nice.org.uk%2Fcovid-19&data=04%7C01%7CRachel.Gledhill%40phe.gov.uk%7C858df0dbbf8b42896a8608d941412c7f%7Cee4e14994a354b2ead475f3cf9de8666%7C0%7C0%7C637612570432156899%7CUnknown%7CTWFpbGZsb3d8eyJWIjoiMC4wLjAwMDAiLCJQIjoiV2luMzIiLCJBTiI6Ik1haWwiLCJXVCI6Mn0%3D%7C1000&sdata=KcZ4Duwg830UNjEdxrd7qwvp9STI4eQJTCXKUvUEUNE%3D&reserved=0) |
| National Institute for Health Research (NIHR) | [https://www.crd.york.ac.uk/prospero/](https://eur01.safelinks.protection.outlook.com/?url=https%3A%2F%2Fwww.crd.york.ac.uk%2Fprospero%2F&data=04%7C01%7CRachel.Gledhill%40phe.gov.uk%7C858df0dbbf8b42896a8608d941412c7f%7Cee4e14994a354b2ead475f3cf9de8666%7C0%7C0%7C637612570432156899%7CUnknown%7CTWFpbGZsb3d8eyJWIjoiMC4wLjAwMDAiLCJQIjoiV2luMzIiLCJBTiI6Ik1haWwiLCJXVCI6Mn0%3D%7C1000&sdata=C8QiDj2s8%2BcrECgw2bSqAlRDkrtqN1kLJv42nQWNzL8%3D&reserved=0) |
| Norwegian Institute of Public Health (NIPH) | [https://www.fhi.no/en/qk/systematic-reviews-hta/map/](https://eur01.safelinks.protection.outlook.com/?url=https%3A%2F%2Fwww.fhi.no%2Fen%2Fqk%2Fsystematic-reviews-hta%2Fmap%2F&data=04%7C01%7CRachel.Gledhill%40phe.gov.uk%7C858df0dbbf8b42896a8608d941412c7f%7Cee4e14994a354b2ead475f3cf9de8666%7C0%7C0%7C637612570432166857%7CUnknown%7CTWFpbGZsb3d8eyJWIjoiMC4wLjAwMDAiLCJQIjoiV2luMzIiLCJBTiI6Ik1haWwiLCJXVCI6Mn0%3D%7C1000&sdata=4CjFUlFRmCqCfxdxGdc9gnnBoooAA6H9w9v9GuXa6kg%3D&reserved=0) |
| Oxford COVID-19 Evidence Service | [https://www.cebm.net/oxford-covid-19-evidence-service/](https://eur01.safelinks.protection.outlook.com/?url=https%3A%2F%2Fwww.cebm.net%2Foxford-covid-19-evidence-service%2F&data=04%7C01%7CRachel.Gledhill%40phe.gov.uk%7C858df0dbbf8b42896a8608d941412c7f%7Cee4e14994a354b2ead475f3cf9de8666%7C0%7C0%7C637612570432166857%7CUnknown%7CTWFpbGZsb3d8eyJWIjoiMC4wLjAwMDAiLCJQIjoiV2luMzIiLCJBTiI6Ik1haWwiLCJXVCI6Mn0%3D%7C1000&sdata=lo8RUUa72qY1VIecOCRSGnHUfMjF005pGw8rK%2BDAgc0%3D&reserved=0) |
| Santé Publique France | [https://www.santepubliquefrance.fr/maladies-et-traumatismes/maladies-et-infections-respiratoires/infection-a-coronavirus/articles/covid-19-etat-des-connaissances-et-veille-documentaire](https://eur01.safelinks.protection.outlook.com/?url=https%3A%2F%2Fwww.santepubliquefrance.fr%2Fmaladies-et-traumatismes%2Fmaladies-et-infections-respiratoires%2Finfection-a-coronavirus%2Farticles%2Fcovid-19-etat-des-connaissances-et-veille-documentaire&data=04%7C01%7CRachel.Gledhill%40phe.gov.uk%7C858df0dbbf8b42896a8608d941412c7f%7Cee4e14994a354b2ead475f3cf9de8666%7C0%7C0%7C637612570432176821%7CUnknown%7CTWFpbGZsb3d8eyJWIjoiMC4wLjAwMDAiLCJQIjoiV2luMzIiLCJBTiI6Ik1haWwiLCJXVCI6Mn0%3D%7C1000&sdata=dCQLzG%2FVx6fjXNZATwaQUAbIHs4vKPn2l9fBHmgvfF0%3D&reserved=0) |
| World Health Organization (WHO) | [https://search.bvsalud.org/global-literature-on-novel-coronavirus-2019-ncov/#](https://eur01.safelinks.protection.outlook.com/?url=https%3A%2F%2Fsearch.bvsalud.org%2Fglobal-literature-on-novel-coronavirus-2019-ncov%2F&data=04%7C01%7CRachel.Gledhill%40phe.gov.uk%7C858df0dbbf8b42896a8608d941412c7f%7Cee4e14994a354b2ead475f3cf9de8666%7C0%7C0%7C637612570432176821%7CUnknown%7CTWFpbGZsb3d8eyJWIjoiMC4wLjAwMDAiLCJQIjoiV2luMzIiLCJBTiI6Ik1haWwiLCJXVCI6Mn0%3D%7C1000&sdata=KBGSvxt%2BUmZ0LW013stMWGGmdh1XuPLTVJNMjcBF5ww%3D&reserved=0) |
| McMaster Forum | [https://www.mcmasterforum.org/find-evidence/products?ProductTypes=Rapid%20evidence%20profile;](https://eur01.safelinks.protection.outlook.com/?url=https%3A%2F%2Fwww.mcmasterforum.org%2Ffind-evidence%2Fproducts%3FProductTypes%3DRapid%2520evidence%2520profile&data=04%7C01%7CRachel.Gledhill%40phe.gov.uk%7C858df0dbbf8b42896a8608d941412c7f%7Cee4e14994a354b2ead475f3cf9de8666%7C0%7C0%7C637612570432176821%7CUnknown%7CTWFpbGZsb3d8eyJWIjoiMC4wLjAwMDAiLCJQIjoiV2luMzIiLCJBTiI6Ik1haWwiLCJXVCI6Mn0%3D%7C1000&sdata=Ksy2OmGONU%2F7%2FhsNJ2YfcZ5AitlqtGNlVmxWsafQHt0%3D&reserved=0) |
| COVID-19 Quick Response Reports for the NL | [https://www.nlcahr.mun.ca/CHRSP/COVIDQuickResponse.php](https://eur01.safelinks.protection.outlook.com/?url=https%3A%2F%2Fwww.nlcahr.mun.ca%2FCHRSP%2FCOVIDQuickResponse.php&data=04%7C01%7CRachel.Gledhill%40phe.gov.uk%7C858df0dbbf8b42896a8608d941412c7f%7Cee4e14994a354b2ead475f3cf9de8666%7C0%7C0%7C637612570432186771%7CUnknown%7CTWFpbGZsb3d8eyJWIjoiMC4wLjAwMDAiLCJQIjoiV2luMzIiLCJBTiI6Ik1haWwiLCJXVCI6Mn0%3D%7C1000&sdata=vpVYgMMCVshfFwNcc5%2BH3N%2BpYZR1%2BXytk8vBad6Nffs%3D&reserved=0) |
| ECDC | [https://www.ecdc.europa.eu/en](https://eur01.safelinks.protection.outlook.com/?url=https%3A%2F%2Fwww.ecdc.europa.eu%2Fen&data=04%7C01%7CRachel.Gledhill%40phe.gov.uk%7C858df0dbbf8b42896a8608d941412c7f%7Cee4e14994a354b2ead475f3cf9de8666%7C0%7C0%7C637612570432186771%7CUnknown%7CTWFpbGZsb3d8eyJWIjoiMC4wLjAwMDAiLCJQIjoiV2luMzIiLCJBTiI6Ik1haWwiLCJXVCI6Mn0%3D%7C1000&sdata=rP%2BRLP6O1LazQ9%2Ba6pFmwq5aVcI19cAzDCQDiw58bQg%3D&reserved=0) |
| PHE Covid-19 Evidence Systematic review updates | Available in 'Keeping up to date' Teams channel, in Systematic Review folder |
| Public Health Wales Observatory, rapid evidence summaries | [http://www.publichealthwalesobservatory.wales.nhs.uk/coronavirus-covid-19-publications](https://eur01.safelinks.protection.outlook.com/?url=http%3A%2F%2Fwww.publichealthwalesobservatory.wales.nhs.uk%2Fcoronavirus-covid-19-publications&data=04%7C01%7CRachel.Gledhill%40phe.gov.uk%7C858df0dbbf8b42896a8608d941412c7f%7Cee4e14994a354b2ead475f3cf9de8666%7C0%7C0%7C637612570432196727%7CUnknown%7CTWFpbGZsb3d8eyJWIjoiMC4wLjAwMDAiLCJQIjoiV2luMzIiLCJBTiI6Ik1haWwiLCJXVCI6Mn0%3D%7C1000&sdata=EyKzkVrjTEx93mYOCEGkelnkumJuNLDZoTl7bE6QQww%3D&reserved=0) |
| New South Wales, Agency for Clinical Innovation, COVID-19 Critical Intelligence Unit | [https://www.aci.health.nsw.gov.au/covid-19/critical-intelligence-unit/evidence-check](https://eur01.safelinks.protection.outlook.com/?url=https%3A%2F%2Fwww.aci.health.nsw.gov.au%2Fcovid-19%2Fcritical-intelligence-unit%2Fevidence-check&data=04%7C01%7CRachel.Gledhill%40phe.gov.uk%7C858df0dbbf8b42896a8608d941412c7f%7Cee4e14994a354b2ead475f3cf9de8666%7C0%7C0%7C637612570432196727%7CUnknown%7CTWFpbGZsb3d8eyJWIjoiMC4wLjAwMDAiLCJQIjoiV2luMzIiLCJBTiI6Ik1haWwiLCJXVCI6Mn0%3D%7C1000&sdata=OsQLh0kS3fQjASnHkh%2BqeN68E4hGj6sQUXb30ycLmdc%3D&reserved=0) |
| NIHR Applied Research Collaboration (ARC) West, COVID-19 rapid reports | [https://arc-w.nihr.ac.uk/research-and-implementation/covid-19-response/](https://eur01.safelinks.protection.outlook.com/?url=https%3A%2F%2Farc-w.nihr.ac.uk%2Fresearch-and-implementation%2Fcovid-19-response%2F&data=04%7C01%7CRachel.Gledhill%40phe.gov.uk%7C858df0dbbf8b42896a8608d941412c7f%7Cee4e14994a354b2ead475f3cf9de8666%7C0%7C0%7C637612570432206686%7CUnknown%7CTWFpbGZsb3d8eyJWIjoiMC4wLjAwMDAiLCJQIjoiV2luMzIiLCJBTiI6Ik1haWwiLCJXVCI6Mn0%3D%7C1000&sdata=ckLKLH%2FF676qR%2Bj1kUe3g%2FJ9QqziHSLzh7xIInLzfRU%3D&reserved=0) |
| Evidence Aid, Coronavirus (COVID-19) collection | [https://evidenceaid.org/evidence/coronavirus-covid-19/](https://eur01.safelinks.protection.outlook.com/?url=https%3A%2F%2Fevidenceaid.org%2Fevidence%2Fcoronavirus-covid-19%2F&data=04%7C01%7CRachel.Gledhill%40phe.gov.uk%7C858df0dbbf8b42896a8608d941412c7f%7Cee4e14994a354b2ead475f3cf9de8666%7C0%7C0%7C637612570432206686%7CUnknown%7CTWFpbGZsb3d8eyJWIjoiMC4wLjAwMDAiLCJQIjoiV2luMzIiLCJBTiI6Ik1haWwiLCJXVCI6Mn0%3D%7C1000&sdata=5Gcpes2n0adpyL9iSgZ5f%2FT2qx%2FEZQhqjnT7xssB0b8%3D&reserved=0) |
| SAHMRI - based Health Policy Centre | [https://www.sahmri.org/covid19/](https://eur01.safelinks.protection.outlook.com/?url=https%3A%2F%2Fwww.sahmri.org%2Fcovid19%2F&data=04%7C01%7CRachel.Gledhill%40phe.gov.uk%7C858df0dbbf8b42896a8608d941412c7f%7Cee4e14994a354b2ead475f3cf9de8666%7C0%7C0%7C637612570432206686%7CUnknown%7CTWFpbGZsb3d8eyJWIjoiMC4wLjAwMDAiLCJQIjoiV2luMzIiLCJBTiI6Ik1haWwiLCJXVCI6Mn0%3D%7C1000&sdata=rrzQGdIRQQR%2Fy8%2FNdeMsddKURUP8s1bKNmT%2BM%2FpxExc%3D&reserved=0) |
| SAGE scientific evidence | [https://www.gov.uk/government/collections/scientific-evidence-supporting-the-government-response-to-coronavirus-covid-19](https://eur01.safelinks.protection.outlook.com/?url=https%3A%2F%2Fwww.gov.uk%2Fgovernment%2Fcollections%2Fscientific-evidence-supporting-the-government-response-to-coronavirus-covid-19&data=04%7C01%7CRachel.Gledhill%40phe.gov.uk%7C858df0dbbf8b42896a8608d941412c7f%7Cee4e14994a354b2ead475f3cf9de8666%7C0%7C0%7C637612570432216643%7CUnknown%7CTWFpbGZsb3d8eyJWIjoiMC4wLjAwMDAiLCJQIjoiV2luMzIiLCJBTiI6Ik1haWwiLCJXVCI6Mn0%3D%7C1000&sdata=F47Q9%2BjifdpDsS4TdXhgZ1XhLzC%2BjcyRLcvR4bsZJoc%3D&reserved=0) |

**Appendix B: Comprehensive search strategy**

**Database: Ovid MEDLINE(R) ALL <1946 to April 05, 2021>**

Date: 05/04/2021

1 exp coronavirus/ (65864)

2 exp Coronavirus Infections/ (79395)

3 ((corona* or corono*) adj1 (virus* or viral* or virinae*)).tw. (1880)

4 (covid or coronavirus* or coronovirus* or coronavirinae* or Coronavirus* or Coronovirus* or Wuhan* or Hubei* or Huanan or "2019-nCoV" or 2019nCoV or nCoV2019 or "nCoV-2019" or "COVID-19" or COVID19 or "CORVID-19" or CORVID19 or "WN-CoV" or WNCoV or "HCoV-19" or HCoV19 or CoV or "2019 novel*" or Ncov or "n-cov" or "SARS-CoV-2" or "SARSCoV-2" or "SARSCoV2" or "SARS-CoV2" or SARSCov19 or "SARS-Cov19" or "SARSCov-19" or "SARS-Cov-19" or Ncovor or Ncorona* or Ncorono* or NcovWuhan* or NcovHubei* or NcovChina* or NcovChinese*).tw. (135936)

5 or/1-4 (146322)

6 exp Cross Infection/ (60912)

7 exp hospital infection/ (60912)

8 exp Health Personnel/ (534520)

9 exp Health Workforce/ (13501)

10 Infectious Disease Transmission, Patient-to-Professional/ (5050)

11 Infectious Disease Transmission, Professional-to-Patient/ (1874)

12 Patients/ (21830)

13 nosocomial.ti,ab,kw. (31450)

14 "hospital acquired".ti,ab,kw. (10306)

15 "healthcare acquired".ti,ab,kw. (297)

16 "health-care acquired".ti,ab,kw. (137)

17 "hospital associated".ti,ab,kw. (1235)

18 "healthcare associated".ti,ab,kw. (5184)

19 "health-care associated".ti,ab,kw. (2602)

20 "hospital onset".ti,ab,kw. (440)

21 "health-care onset".ti,ab,kw. (6)

22 "healthcare onset".ti,ab,kw. (16)

23 "hospital transmission".ti,ab,kw. (203)

24 "health-care transmission".ti,ab,kw. (14)

25 "healthcare transmission".ti,ab,kw. (10)

26 "hospital team*".ti,ab,kw. (420)

27 (HW or HWs or HCW or HCWs or HCP or HCPs).ti,ab,kw. (14658)

28 "healthcare workforce".ti,ab,kw. (656)

29 "health-care workforce".ti,ab,kw. (797)

30 "healthcare team*".ti,ab,kw. (2818)

31 "health-care team*".ti,ab,kw. (4841)

32 "healthcare worker*".ti,ab,kw. (13018)

33 "health-care worker*".ti,ab,kw. (14681)

34 "healthcare practitioner*".ti,ab,kw. (1859)

35 "health-care practitioner*".ti,ab,kw. (2485)

36 "healthcare professional*".ti,ab,kw. (26020)

37 "health-care professional*".ti,ab,kw. (25925)

38 "health staff".ti,ab,kw. (2393)

39 "healthcare staff".ti,ab,kw. (1959)

40 "health-care staff".ti,ab,kw. (1433)

41 "health personnel".ti,ab,kw. (7626)

42 "healthcare personnel".ti,ab,kw. (2046)

43 "health-care personnel".ti,ab,kw. (2686)

44 "hospital personnel".ti,ab,kw. (2046)

45 "medical staff".ti,ab,kw. (12693)

46 "non-medical staff".ti,ab,kw. (220)

47 "nursing staff".ti,ab,kw. (12667)

48 "hospital staff".ti,ab,kw. (5606)

49 "clinical staff".ti,ab,kw. (3452)

50 "non-clinical staff".ti,ab,kw. (205)

51 "general hospital*".ti,ab,kw. (39606)

52 "acute hospital".ti,ab,kw. (3704)

53 "intra-hospital".ti,ab,kw. (605)

54 Secondary Care Centers/ (253)

55 Secondary Care/ (697)

56 (secondary adj2 (care or healthcare or health-care or service or center or centre)).ti,ab,kw. (9970)

57 Tertiary Care Centers/ (15548)

58 Tertiary Healthcare/ (1206)

59 (tertiary adj2 (care or healthcare or health-care or service or center or centre)).ti,ab,kw. (78689)

60 Cancer Care Facilities/ (5581)

61 or/6-60 (870160)

62 infection control/ (26779)

63 patient isolation/ (4274)

64 quarantine/ (4392)

65 Contact Tracing/ (5113)

66 mandatory testing/ (852)

67 mass screening/ (106723)

68 exp hand hygiene/ (7320)

69 Hand Sanitizers/ (225)

70 exp Sterilization/ (31599)

71 population surveillance/ (60705)

72 Occupational Diseases/pc (17211)

73 sanitation/ (7535)

74 universal precautions/ (1659)

75 decontamination/ (5106)

76 Air Conditioning/ (2733)

77 Ventilation/ (5894)

78 Cross infection/ (57976)

79 Disease Transmission, Infectious/pc (4906)

80 Occupational Exposure/pc (7186)

81 control*.ti,ab,kw. (4024794)

82 prevent*.ti,ab,kw. (1497390)

83 protect*.ti,ab,kw. (847158)

84 precaution*.ti,ab,kw. (23456)

85 test*.ti,ab,kw. (3360107)

86 screen*.ti,ab,kw. (799826)

87 cohort*.ti,ab,kw. (654680)

88 barrier*.ti,ab,kw. (318568)

89 roster*.ti,ab,kw. (1715)

90 ((healthcare or workforce or service*) adj3 reconfigur*).ti,ab,kw. (243)

91 isolat*.ti,ab,kw. (1379830)

92 quarantin*.ti,ab,kw. (7978)

93 segregat*.ti,ab,kw. (76114)

94 hygien*.ti,ab,kw. (76604)

95 "hand wash*".ti,ab,kw. (3041)

96 handwash*.ti,ab,kw. (2391)

97 ventilation.ti,ab,kw. (126110)

98 ventilate.ti,ab,kw. (1298)

99 ventilated.ti,ab,kw. (29639)

100 "air flow*".ti,ab,kw. (4711)

101 (air adj2 circulat*).ti,ab,kw. (411)

102 "air condition*".ti,ab,kw. (3700)

103 sanitis*.ti,ab,kw. (214)

104 sanitiz*.ti,ab,kw. (2834)

105 disinfect*.ti,ab,kw. (31166)

106 sterilis*.ti,ab,kw. (3475)

107 steriliz*.ti,ab,kw. (34194)

108 decontaminat*.ti,ab,kw. (13114)

109 irradiation.ti,ab. (200351)

110 (patient* adj2 cohort*).ti,ab,kw. (59120)

111 (staff adj2 cohort*).ti,ab,kw. (72)

112 "social distanc*".ti,ab,kw. (5285)

113 "social* distan*".ti,ab,kw. (5354)

114 "physical distanc*".ti,ab,kw. (1991)

115 (visitor* adj2 restrict*).ti,ab,kw. (99)

116 triag*.ti,ab,kw. (22358)

117 "risk assessment*".ti,ab,kw. (74709)

118 (reduc* adj2 contact).ti,ab,kw. (2060)

119 or/62-118 (10443358)

120 5 and 61 and 119 (9767)

121 limit 120 to yr="2020 -Current" (8670)

**Database: Embase <1974 to 2021 April 05>**

Date: 05/04/2021

1 exp Coronavirinae/ (23603)

2 exp Coronavirus Infection/ (24618)

3 "coronavirus disease 2019".sh. (100075)

4 ((corona* or corono*) adj1 (virus* or viral* or virinae*)).ti,ab,kw. (2569)

5 (covid or coronavirus* or coronovirus* or coronavirinae* or Coronavirus* or Coronovirus* or Wuhan* or Hubei* or Huanan or "2019-nCoV" or 2019nCoV or nCoV2019 or "nCoV-2019" or "COVID-19" or COVID19 or "CORVID-19" or CORVID19 or "WN-CoV" or WNCoV or "HCoV-19" or HCoV19 or CoV or "2019 novel*" or Ncov or "n-cov" or "SARS-CoV-2" or "SARSCoV-2" or "SARSCoV2 r SARS-CoV2" or SARSCov19 or "SARS-Cov19" or "SARSCov-19" or "SARS-Cov-19" or Ncovor or Ncorona* or Ncorono* or NcovWuhan* or NcovHubei* or NcovChina* or NcovChinese*).ti,ab,kw. (138841)

6 or/1-5 (155899)

7 exp Cross Infection/ (19892)

8 exp hospital infection/ (48529)

9 exp Health Care Personnel/ (1656621)

10 disease transmission/ (102681)

11 Patients/ (1057831)

12 nosocomial.ti,ab,kw. (44917)

13 "hospital acquired".ti,ab,kw. (15983)

14 "healthcare acquired".ti,ab,kw. (529)

15 "health-care acquired".ti,ab,kw. (228)

16 "hospital associated".ti,ab,kw. (1844)

17 "healthcare associated".ti,ab,kw,sh. (8628)

18 "health-care associated".ti,ab,kw. (3829)

19 "hospital onset".ti,ab,kw. (733)

20 "health-care onset".ti,ab,kw. (8)

21 "healthcare onset".ti,ab,kw. (33)

22 "hospital transmission".ti,ab,kw. (270)

23 "health-care transmission".ti,ab,kw. (13)

24 "healthcare transmission".ti,ab,kw. (12)

25 "hospital team*".ti,ab,kw. (707)

26 (HW or HWs or HCW or HCWs or HCP or HCPs).ti,ab,kw. (21117)

27 "healthcare workforce".ti,ab,kw. (772)

28 "health-care workforce".ti,ab,kw. (825)

29 "healthcare team*".ti,ab,kw. (4760)

30 "health-care team*".ti,ab,kw. (6513)

31 "healthcare worker*".ti,ab,kw. (16860)

32 "health-care worker*".ti,ab,kw. (18060)

33 "healthcare practitioner*".ti,ab,kw. (2568)

34 "health-care practitioner*".ti,ab,kw. (3160)

35 "healthcare professional*".ti,ab,kw. (39001)

36 "health-care professional*".ti,ab,kw. (35115)

37 "health staff".ti,ab,kw. (3118)

38 "healthcare staff".ti,ab,kw. (2625)

39 "health-care staff".ti,ab,kw. (1834)

40 "health personnel".ti,ab,kw. (6962)

41 "healthcare personnel".ti,ab,kw. (2598)

42 "health-care personnel".ti,ab,kw. (3413)

43 "hospital personnel".ti,ab,kw. (2363)

44 "medical staff".ti,ab,kw. (19219)

45 "non-medical staff".ti,ab,kw. (356)

46 "nursing staff".ti,ab,kw. (19819)

47 "hospital staff".ti,ab,kw. (7843)

48 "clinical staff".ti,ab,kw. (5807)

49 "non-clinical staff".ti,ab,kw. (318)

50 "general hospital*".ti,ab,kw. (59707)

51 "acute hospital".ti,ab,kw. (5986)

52 "intra-hospital".ti,ab,kw. (1355)

53 exp secondary health care/ (8778)

54 (secondary adj2 (care or healthcare or health-care or service or center or centre)).ti,ab,kw. (16892)

55 exp tertiary health care/ (111389)

56 (tertiary adj2 (care or healthcare or health-care or service or center or centre)).ti,ab,kw. (140705)

57 or/7-56 (2984780)

58 exp Medical Device Contamination/ (1029)

59 exp Health Care Personnel Management/ (3200)

60 exp patient isolation/ (1614)

61 social distance/ (4184)

62 exp quarantine/ (5969)

63 exp infection prevention/ (66200)

64 exp infection control/ (110353)

65 universal precaution/ (492)

66 exp Sterilization/ (20479)

67 population surveillance/ (175183)

68 Occupational Diseases/pc (5245)

69 sanitation/ (14668)

70 exp ultraviolet irradiation/ (15880)

71 "point of care testing"/ (15123)

72 mandatory testing/ (900)

73 Mass screening/ (56690)

74 disease transmission/pc (3315)

75 occupational exposure/pc (2690)

76 "prevention and control".sh. (29011)

77 control*.ti,ab,kw. (5318676)

78 prevent*.ti,ab,kw. (1975590)

79 protect*.ti,ab,kw. (1084382)

80 precaution*.ti,ab,kw. (31894)

81 test*.ti,ab,kw. (4598162)

82 screen*.ti,ab,kw. (1139071)

83 cohort*.ti,ab,kw. (1117994)

84 barrier*.ti,ab,kw. (403524)

85 roster*.ti,ab,kw. (2053)

86 ((healthcare or workforce or service*) adj3 reconfigur*).ti,ab,kw. (391)

87 isolat*.ti,ab,kw. (1649434)

88 quarantin*.ti,ab,kw. (7784)

89 segregat*.ti,ab,kw. (86626)

90 hygien*.ti,ab,kw. (92809)

91 "hand wash*".ti,ab,kw. (4280)

92 handwash*.ti,ab,kw. (2876)

93 ventilation.ti,ab,kw. (191570)

94 ventilate.ti,ab,kw. (1942)

95 ventilated.ti,ab,kw. (44302)

96 "air flow*".ti,ab,kw. (6881)

97 (air adj2 circulat*).ti,ab,kw. (598)

98 "air condition*".ti,ab,kw. (4786)

99 sanitis*.ti,ab,kw. (292)

100 sanitiz*.ti,ab,kw. (3387)

101 disinfect*.ti,ab,kw,sh. (51305)

102 sterilis*.ti,ab,kw. (4797)

103 steriliz*.ti,ab,kw. (34700)

104 decontaminat*.ti,ab,kw. (16867)

105 irradiation.ti,ab. (235891)

106 (patient* adj2 cohort*).ti,ab,kw. (123873)

107 (staff adj2 cohort*).ti,ab,kw. (115)

108 "social* distan*".ti,ab,kw,sh. (10450)

109 "physical distanc*".ti,ab,kw. (1992)

110 (visitor* adj2 restrict*).ti,ab,kw. (128)

111 triag*.ti,ab,kw. (35433)

112 "risk assessment*".ti,ab,kw. (104524)

113 (reduc* adj2 contact).ti,ab,kw. (2353)

114 or/58-113 (13637840)

115 6 and 57 and 114 (19835)

116 limit 115 to yr="2020 -Current" (17036)

**Cochrane Central Register of Controlled Trials (CENTRAL)**

Date: 06/04/2021

ID Search Hits

#1 MeSH descriptor: [Coronavirus] explode all trees 242

#2 MeSH descriptor: [Coronavirus Infections] explode all trees 789

#3 (((corona* or corono*) NEXT (virus* or viral* or virinae*))):ti,ab,kw 212

#4 ((covid or coronavirus* or coronovirus* or coronavirinae* or Coronavirus* or Coronovirus* or Wuhan* or Hubei* or Huanan or "2019-nCoV" or 2019nCoV or nCoV2019 or "nCoV-2019" or "COVID-19" or COVID19 or "CORVID-19" or CORVID19 or "WN-CoV" or WNCoV or "HCoV-19" or HCoV19 or CoV or "2019 novel*" or Ncov or "n-cov" or "SARS-CoV-2" or "SARSCoV-2" or "SARSCoV2" or "SARS-CoV2" or SARSCov19 or "SARS-Cov19" or "SARSCov-19" or "SARS-Cov-19" or Ncovor or Ncorona* or Ncorono* or NcovWuhan* or NcovHubei* or NcovChina* or NcovChinese*)):ti,ab,kw 5519

#5 {OR #1-#4} 5550

#6 MeSH descriptor: [Cross Infection] explode all trees 1471

#7 MeSH descriptor: [Health Personnel] explode all trees 9107

#8 MeSH descriptor: [Health Workforce] explode all trees 22

#9 MeSH descriptor: [Infectious Disease Transmission, Patient-to-Professional] this term only 72

#10 MeSH descriptor: [Infectious Disease Transmission, Professional-to-Patient] this term only 31

#11 MeSH descriptor: [Patients] explode all trees 2698

#12 (nosocomial):ti,ab,kw 1699

#13 ("hospital acquired"):ti,ab,kw 810

#14 ("healthcare acquired"):ti,ab,kw 23

#15 ("health-care acquired"):ti,ab,kw 5

#16 ("hospital associated"):ti,ab,kw 94

#17 ("healthcare associated"):ti,ab,kw 336

#18 ("health-care associated"):ti,ab,kw 150

#19 ("hospital onset"):ti,ab,kw 9

#20 ("health-care onset"):ti,ab,kw 2

#21 ("healthcare onset"):ti,ab,kw 2

#22 ("hospital transmission"):ti,ab,kw 5

#23 ("health-care transmission"):ti,ab,kw 0

#24 ("healthcare transmission"):ti,ab,kw 0

#25 (hospital NEXT team*):ti,ab,kw 46

#26 (HW or HWs or HCW or HCWs or HCP or HCPs):ti,ab,kw 988

#27 ("healthcare workforce"):ti,ab,kw 22

#28 ("health-care workforce"):ti,ab,kw 6

#29 (healthcare NEXT team*):ti,ab,kw 279

#30 (health-care NEXT team*):ti,ab,kw 329

#31 (healthcare NEXT worker*):ti,ab,kw 954

#32 (health-care NEXT worker*):ti,ab,kw 806

#33 (healthcare NEXT practitioner*):ti,ab,kw 108

#34 (health-care NEXT practitioner*):ti,ab,kw 132

#35 (healthcare NEXT professional*):ti,ab,kw 2235

#36 (health-care NEXT professional*):ti,ab,kw 1716

#37 ("health staff"):ti,ab,kw 224

#38 ("healthcare staff"):ti,ab,kw 188

#39 ("health-care staff"):ti,ab,kw 122

#40 ("health personnel"):ti,ab,kw 3654

#41 ("healthcare personnel"):ti,ab,kw 151

#42 ("health-care personnel"):ti,ab,kw 3250

#43 ("hospital personnel"):ti,ab,kw 311

#44 ("medical staff"):ti,ab,kw 1452

#45 ("non-medical staff"):ti,ab,kw 10

#46 ("nursing staff"):ti,ab,kw 2275

#47 ("hospital staff"):ti,ab,kw 425

#48 ("clinical staff"):ti,ab,kw 681

#49 ("non-clinical staff"):ti,ab,kw 19

#50 (general NEXT hospital*):ti,ab,kw 3294

#51 (acute NEXT hospital*):ti,ab,kw 738

#52 ("intra-hospital"):ti,ab,kw 64

#53 MeSH descriptor: [Secondary Care Centers] this term only 7

#54 MeSH descriptor: [Secondary Care] this term only 40

#55 ((secondary NEAR/2 (care or healthcare or health-care or service or center or centre))):ti,ab,kw 2612

#56 MeSH descriptor: [Tertiary Care Centers] this term only 335

#57 MeSH descriptor: [Tertiary Healthcare] this term only 20

#58 ((tertiary NEAR/2 (care or healthcare or health-care or service or center or centre))):ti,ab,kw 8411

#59 MeSH descriptor: [Cancer Care Facilities] this term only 90

#60 {OR #6-#59} 42378

#61 MeSH descriptor: [Infection Control] this term only 548

#62 MeSH descriptor: [Patient Isolation] explode all trees 54

#63 MeSH descriptor: [Quarantine] this term only 12

#64 MeSH descriptor: [Contact Tracing] explode all trees 82

#65 MeSH descriptor: [Mandatory Testing] explode all trees 3

#66 MeSH descriptor: [Mass Screening] explode all trees 3839

#67 MeSH descriptor: [Hand Hygiene] explode all trees 458

#68 MeSH descriptor: [Hand Sanitizers] explode all trees 37

#69 MeSH descriptor: [Sterilization] explode all trees 491

#70 MeSH descriptor: [Population Surveillance] explode all trees 518

#71 MeSH descriptor: [Occupational Diseases] this term only 884

#72 MeSH descriptor: [Sanitation] explode all trees 251

#73 MeSH descriptor: [Universal Precautions] explode all trees 16

#74 MeSH descriptor: [Decontamination] explode all trees 82

#75 MeSH descriptor: [Air Conditioning] explode all trees 34

#76 MeSH descriptor: [Ventilation] explode all trees 83

#77 MeSH descriptor: [Cross Infection] explode all trees 1471

#78 MeSH descriptor: [Disease Transmission, Infectious] this term only and with qualifier(s): [prevention & control - PC] 94

#79 MeSH descriptor: [Occupational Exposure] this term only and with qualifier(s): [prevention & control - PC] 125

#80 (control*):ti,ab,kw 1025915

#81 (prevent*):ti,ab,kw 234524

#82 (protect*):ti,ab,kw 42045

#83 (precaution*):ti,ab,kw 1420

#84 (test*):ti,ab,kw 389392

#85 (screen*):ti,ab,kw 76223

#86 (cohort*):ti,ab,kw 59292

#87 (barrier*):ti,ab,kw 19053

#88 (roster*):ti,ab,kw 103

#89 (((healthcare or workforce or service*) NEAR/3 reconfigur*)):ti,ab,kw 15

#90 (isolat*):ti,ab,kw 27744

#91 (quarantin*):ti,ab,kw 217

#92 (segregat*):ti,ab,kw 465

#93 (hygien*):ti,ab,kw 9132

#94 (hand NEXT wash*):ti,ab,kw 737

#95 (handwash*):ti,ab,kw 518

#96 (ventilation):ti,ab,kw 27488

#97 (ventilate):ti,ab,kw 222

#98 (ventilated):ti,ab,kw 5405

#99 (air NEXT flow*):ti,ab,kw 319

#100 ((air NEAR/2 circulat*)):ti,ab,kw 28

#101 (air NEXT condition*):ti,ab,kw 860

#102 (sanitis*):ti,ab,kw 14

#103 (sanitiz*):ti,ab,kw 187

#104 (disinfect*):ti,ab,kw 2511

#105 (sterilis*):ti,ab,kw 409

#106 (steriliz*):ti,ab,kw 2334

#107 (decontaminat*):ti,ab,kw 855

#108 (irradiation):ti,ab,kw 8411

#109 ((patient* NEAR/2 cohort*)):ti,ab,kw 6423

#110 ((staff NEAR/2 cohort*)):ti,ab,kw 7

#111 (social* NEXT distanc*):ti,ab,kw 301

#112 (physical* NEXT distanc*):ti,ab,kw 53

#113 ((visitor* NEAR/2 restrict*)):ti,ab,kw 5

#114 (triag*):ti,ab,kw 1849

#115 (risk NEXT assessment*):ti,ab,kw 25789

#116 ((reduc* NEAR/2 contact)):ti,ab,kw 209

#117 {OR #61-#116} 1224473

#118 #5 AND #60 AND #117 483

#119 Date limit 2020-2021 445

[**Covid-19 Portfolio**](https://icite.od.nih.gov/covid19/search/#home)

**(Sources: medRxiv, bioRxiv, arXiv, ChemRxiv, Research Square, SSRN, Preprints.org, Qeios)**

Date: 07/04/2021

(nosocomial OR "hospital acquired" OR "healthcare acquired" OR "health-care acquired" OR "hospital associated" OR "healthcare associated" OR "hospital associated" OR "healthcare associated" OR "health-care associated" OR "hospital onset" OR "health-care onset" OR "healthcare onset" OR "hospital transmission"~2 OR "health-care transmission" OR "healthcare transmission" OR "hospital team" OR "hospital teams" OR "healthcare workforce" OR "health-care workforce" OR "healthcare team" OR "healthcare teams" OR "health-care team" OR "health-care teams" OR "healthcare worker" OR "healthcare workers" OR "health-care worker" OR "health-care workers" OR "healthcare practitioner" OR "healthcare practitioners" OR "health-care practitioner" OR "health-care practitioners" OR "healthcare professional" OR "healthcare professionals" OR "health-care professional" OR "health-care professionals" OR "health staff" OR "healthcare staff" OR "health-care staff" OR "health personnel" OR "healthcare personnel" OR "health-care personnel" OR "hospital personnel" OR "medical staff" OR "non-medical staff" OR "nursing staff" OR "hospital staff" OR "clinical staff" OR "non-clinical staff" OR HW OR HWs OR HCW OR HCWs OR HCP OR HCPs OR "general hospital" OR "acute hospital" OR "intra-hospital" OR "secondary care"~2 OR "secondary healthcare"~2 OR "secondary health-care"~2 OR "tertiary care"~2 OR "tertiary healthcare"~2 OR "tertiary health-care"~2) AND (control* OR prevent* OR protect* OR precaution* OR test* OR screen* OR cohort* OR barrier* OR roster* OR isolat* OR quarantin* OR segregat* OR hygien* OR triag* OR "healthcare reconfiguration"~2 OR "service reconfiguration"~2 OR "hand wash" OR handwash* OR ventilation OR ventilate OR ventilated OR "air flow" OR "air circulation" OR "air conditioning" OR sanitis* OR sanitiz* OR disinfect* OR sterilis* OR steriliz* OR decontaminat* OR irradiation OR "social distance" OR "social distancing" OR "socially distant" OR "physical distance" OR "physical distancing" OR "visitor restrictions" OR "restricting visitors"~2 OR "staff cohort" OR "staff cohorts"~2 OR "staff cohorting"~2 OR "patient cohort" OR "patients cohort"~2 OR "patient cohorts"~2 OR "patients cohorts"~2 OR "patient cohorting" OR "risk assessment" OR "risk assessments" OR "reducing contact" OR "reduce contact" OR "reduced contact" OR "contact tracing")

**Search fields: Title, Abstract**

**Total results: 2084**

**Web of Science**

Date: 07/04/2021

(covid or coronavirus* OR "2019-nCoV" or 2019nCoV or nCoV2019 or "nCoV-2019" or "COVID-19" or COVID19 or "CORVID-19" or CORVID19 or "WN-CoV" or WNCoV or "HCoV-19" or HCoV19 or CoV or "2019 novel*" or Ncov or "n-cov" or "SARS-CoV-2" or "SARSCoV-2" or "SARSCoV2 r SARS-CoV2" or SARSCov19 or "SARS-Cov19" or "SARSCov-19" or "SARS-Cov-19" or Ncovor or Ncorona* or Ncorono* or NcovWuhan* or NcovHubei* or NcovChina* or NcovChinese*) AND (nosocomial OR "hospital acquired" OR "healthcare acquired" OR "health-care acquired" OR "hospital associated" OR "healthcare associated" OR "hospital associated" OR "healthcare associated" OR "health-care associated" OR "hospital onset" OR "health-care onset" OR "healthcare onset" OR "hospital transmission" OR "health-care transmission" OR "healthcare transmission" OR "hospital team" OR "hospital teams" OR "healthcare workforce" OR "health-care workforce" OR "healthcare team" OR "healthcare teams" OR "health-care team" OR "health-care teams" OR "healthcare worker" OR "healthcare workers" OR "health-care worker" OR "health-care workers" OR "healthcare practitioner" OR "healthcare practitioners" OR "health-care practitioner" OR "health-care practitioners" OR "healthcare professional" OR "healthcare professionals" OR "health-care professional" OR "health-care professionals" OR "health staff" OR "healthcare staff" OR "health-care staff" OR "health personnel" OR "healthcare personnel" OR "health-care personnel" OR "hospital personnel" OR "medical staff" OR "non-medical staff" OR "nursing staff" OR "hospital staff" OR "clinical staff" OR "non-clinical staff" OR "general hospital" OR "acute hospital" OR "intra-hospital" OR "secondary care" OR (secondary AND (healthcare OR health-care OR "health care")) OR "tertiary care" OR (tertiary AND healthcare OR health-care OR "health care")) AND (control* OR prevent* OR protect* OR precaution* OR test* OR screen* OR cohort* OR barrier* OR roster* OR isolat* OR quarantin* OR segregat* OR hygien* OR triag* OR "healthcare reconfiguration" OR "service reconfiguration" OR "hand wash" OR handwash* OR ventilation OR ventilate OR ventilated OR "air flow" OR "air circulation" OR "air conditioning" OR sanitis* OR sanitiz* OR disinfect* OR sterilis* OR steriliz* OR decontaminat* OR irradiation OR "social distance" OR "social distancing" OR "socially distant" OR "physical distance" OR "physical distancing" OR "visitor restrictions" OR "restricting visitors" OR "staff cohort*" OR "cohorting staff" OR "cohorts of staff" OR "patient cohort" OR "patient* cohort*" OR "patient cohorting" OR "risk assessment" OR "risk assessments" OR "reducing contact" OR "reduce contact" OR "reduced contact" OR "contact tracing")

Date: 2020-2021

**6923 results**

**Appendix C: Extended table of details of studies**

Table 1 – Extended version with all characteristics of included studies

| Study | Country | Publication status | Type of study | No. of participants | Age | % female | Co-morbidities | Hospital setting | Baseline IPC measures | Type of intervention | Intervention description | Duration of follow up | Control | Primary outcome | Secondary outcome |
| --- | --- | --- | --- | --- | --- | --- | --- | --- | --- | --- | --- | --- | --- | --- | --- |
| Abella 2020 | USA | Published | RCT | 132 | median: 33 years (range, 20-66) | 69% | Asthma (17%), Diabetes (3%), Hypertension (21%) | Emergency department, dedicated COVID-19 units | Use of PPE (including masks, eyewear, and gowns) as well as patient screening for COVID-19 symptoms | Pre-exposure prophylaxis | hydroxychloroquine 200-mg tablets, 3 tablets once a day | 8 wks | Placebo | rate of conversion to SARS-CoV-2 positive status via NP RT-PCR after 8 weeks of treatment | adverse event rate; rate of serologic antibody positivity for either nucleocapsid or spike protein antigens; ECG changes after 4 weeks of treatment; clinical outcomes for any partici- pants who became SARS-CoV-2 positive and/or developed COVID-19 symptoms within study period. |
| Chahla, 2021 | Argentina | Pre-print | RCT | 234 | median 38 years (min: 22; max: 69) | 57.30% | Hypertension (9%), Diabetes (7%), Obesity (12%), >60 years (4%), Renal (2%) | Healthcare centres | standard biosecurity care and personal protective equipment (PPE). | Pre-exposure prophylaxis | ivermectin(2 tablets of 6 mg mg weekly) and Iota-Carrageenan (6 sprays per day) | 4 wks | Standard biosecurity care and personal protective equipment (PPE). | reduction in COVID-19 disease rate, measured by RT-PCR | Reduction in presence of COVID-19 symptoms; protection against the appearance of severe stages for COVID-19 disease |
| Grau-Pujol, 2021 | Spain | Pre-print | RCT | 269 | median: 39 years (IQR: 30–50 years) | 73% | Diabetes (0.4%), hypertension (1.9%), chronic respiratory condition (2.6%), other (27.9%) | Hospital, specific unit unclear. | 83% always used COVID-19 recommended PPE at work during the last 20 days | Pre-exposure prophylaxis | hydroxychloroquine (2 tablets of 200 mg daily for first 4 days, then 400mg weekly) | 6 mos | Placebo | incidence of compatible symptoms with COVID-19 with seroconversion or a positive RT-PCR between study arms | the SARS-CoV-2 seroconversion in study arms in both asymptomatic and symptomatic participants; adverse events (AE) related to hydroxychloroquine treatment; incidence of SARS-CoV-2 infection in placebo group ; risk ratio for the different clinical, analytical and microbiological conditions to develop COVID-19. |
| Gupta, 2021 | India | Pre-print | RCT | 199 | Intervention: mean: 32.1 (SD:7.4); Control: mean: 33.6 (SD: 8.6) | Intervention: 40.8% out of 98, Control: 50.5% out of 95 | Malnourished (3.1%) | COVID-19 isolation ward | Standard Preventive Regimen as per institutional guidelines and based on roles | Pre-exposure prophylaxis | Chyawanprash (12 g twice daily) | 30 days | Standard preventive regimen | incidence of COVID-19 cases in both groups confirmed by RT-PCR | comparing the biochemical and hematological parameters before and after the study and through occurrence of any adverse drug reactions; assessment of efficacy of Chyawanprash in preventing other infective diseases through incidence of symptoms; evaluation of effect of Chyawanprash on immunoglobulins and inflammatory markers through comparing the levels of IgG, IgM, IgE, high sensitivity C-Reactive Protein (hsCRP), Tumor Necrosing Factor alpha (TNF alpha) and Interleukins viz., IL-6 and IL-10. |
| Mikhaylov, 2021 | Russia | Pre-print | RCT | 50 | mean: 40.6(SD: 7.6) | 58% | Hypertensive (6%); Hypercholesterolemia (4%) | emergency departments where patients with confirmed/suspected COVID-19 were admitted, intensive care units, and clinical departments | PPE as prescribed by WHO recommendations and local instructions. PPE included respirators class FFP2 or FFP3, full skin covering, and protective eyeglasses. | Pre-exposure prophylaxis | Bromhexine hydrochloride treatment (8 mg 3 times per day) | 8 wks | Standard care | positive nasopharyngeal swab SARS-CoV-2 PCR test or the presence of clinical symptoms of infection within 28 days and during the weeks 5 - 8 after the last contact to subjects with COVID-19 | time from the first contact with a person with suspected/confirmed COVID-19 to the appearance of respiratory infection symptoms; number of days before first positive SARS-CoV-2 test;number of asymptomatic participants with a positive nasopharyngeal swab test; the number of mild, moderate and severe COVID-19 cases; |
| Rajasingham, 2020 | Canada, USA | Published | RCT | 1483 | median age: 41 years (interquar- tile range [IQR], 34 to 49) | 51% | Hypertension(14%), asthma(10%), | emergency department or intensive care unit, on a dedicated COVID-19 hospital ward | Mask/ faceshield use reported over 80% in all groups | Pre-exposure prophylaxis | Hydroxychlroquine(400 mg (2 200-mg tablets) twice separated by 6–8 hours followed by (1) 400 mg (2 200-mg tablets) once weekly or (2) 400 mg (2 200-mg tablets) twice weekly | 12 wks | Placebo | COVID-19–free survival time by PCR confirmed or probable compatible illness. | incidence of confirmed SARS-CoV-2 detection; incidence of possible COVID-19; incidence of hospitalization, death, or other adverse events. |
| Hafeez, 2020 | Pakistan | Pre-print | non-RT | 60 | Intervention: Min-max(20-35); Control: Min-max(20-38) | 28.30% | Anxiety(93.3%) | Entrance of the hospital | Standard PPE (did not specify what types) | Audio-visual triage | glass barrier sheet at triage desk at a distance of more than 6 feet from patient desk, both desks connected with non-touchable mic system for communication | 1 wk | visual triage (outside at entry door) | Anxiety levels | COVID-19 PCR results |
